# The mediation of resilience between physical activity, parenting style, and social anxiety among Chinese adolescents: evidence from a structural equation model

**DOI:** 10.3389/fpsyg.2026.1852448

**Published:** 2026-07-14

**Authors:** Yijie Shi, Li Zhuang, Zhi Gao, Runjun Lv, Mariusz Lipowski

**Affiliations:** 1 Gdansk University of Physical Education and Sport, Gdańsk, Poland; 2 Guangdong Vocational College of Science and Technology, Zhuhai, China; 3 Zhongshan University Affiliated Houhuan Primary School in the High-tech Zone of Zhuhai City, Zhuhai, China; 4 WSB Merito University in Gdańsk, Gdańsk, Poland

**Keywords:** mediating effect, parenting style, physical activity, resilience, social anxiety, structural equation model

## Abstract

**Background:**

Adolescent social anxiety is a common mental health problem. Although physical activity and parenting style have both been linked to anxiety, their joint associations with social anxiety and the mediating role of resilience remain unclear. This study tested an integrated model of physical activity, parenting style, resilience, and social anxiety in Chinese adolescents.

**Methods:**

A total of 1,094 adolescents completed measures of physical activity, parenting style, resilience, and social anxiety. Structural equation modelling was used to examine direct and indirect associations among these variables, with resilience specified as a mediator.

**Results:**

Physical activity and parental emotional warmth were positively associated with resilience and negatively associated with social anxiety. Parental rejection was positively associated with social anxiety but was not significantly related to resilience. Parental overprotection was positively associated with resilience, whereas its direct association with social anxiety was not significant. Resilience was negatively associated with social anxiety and significantly mediated the effects of physical activity, emotional warmth, and overprotection on social anxiety.

**Conclusion:**

Adolescent social anxiety appears to be shaped by both behavioural and family factors, with resilience serving as an important psychological mechanism. These findings support integrated interventions that combine physical activity promotion, supportive parenting, and resilience enhancement.

## 1 Introduction

Adolescent anxiety is a major public-health concern because it is common, often begins early, and contributes to disability during a sensitive developmental period (Kessler et al., 2005; Beesdo et al., 2009). Meta-analytic evidence on age-of-onset distributions suggests that about one third of mental disorders begin before age 14 and nearly half begin before age 18 (Solmi et al., 2022). Community studies further estimate that mental disorders affect a sizeable proportion of children and adolescents worldwide (Bor et al., 2014; Polanczyk et al., 2015). During the first year of the COVID-19 pandemic, pooled prevalence of clinically elevated anxiety symptoms in youth was about 20.5% (Racine et al., 2021), and global modelling estimated a substantial pandemic-associated increase in anxiety burden across countries (Santomauro et al., 2021).

### 1.1 Physical activity and adolescent anxiety

Physical activity (PA) is a modifiable health behaviour that may protect against anxiety (Stubbs et al., 2018; Schuch et al., 2019). Prospective evidence is informative for prevention (McDowell et al., 2019; Kandola et al., 2020). In a systematic review and meta-analysis of cohort studies, higher baseline PA was associated with lower odds of later anxiety symptoms and anxiety disorders, although heterogeneity in PA measurement and covariate adjustment was highlighted (McDowell et al., 2019). These findings support PA as a plausible behavioural target for adolescent anxiety prevention (Bélair et al., 2018; Kandola et al., 2020). In Chinese student samples, regular physical activity has also been linked to lower negative emotion and to better sleep and fewer stress-related problems, with exercise type and dose shaping the association (Yang et al., 2021; Wang et al., 2024b).

Intervention evidence is broadly supportive but methodologically uneven (Aumer and Vögele, 2025; Zhang et al., 2025). A systematic review and meta-analysis of 22 randomized controlled trials reported a moderate reduction in state anxiety after PA interventions compared with minimal or no-intervention controls, while noting small samples, risk of bias, and limited evidence in clinically anxious youth (Aumer and Vögele, 2025). Proposed pathways include distraction from worry, strengthened coping self-efficacy, and habituation to interoceptive sensations, suggesting that individual motivation and social context may shape intervention response (Bouchard et al., 2004; Smith and Merwin, 2021; Teraoka, 2025).

### 1.2 Parenting style and adolescent anxiety

Parenting style provides a salient family context for adolescent anxiety (Pinquart, 2017; Howard et al., 2025). In widely used dimensions, rejection reflects hostility and lack of acceptance, emotional warmth reflects support and affection, and overprotection reflects intrusive control and restricted autonomy (Bruysters and Pilkington, 2023; Dong et al., 2024). Meta-analytic evidence indicates that parenting is related to child anxiety, with control restriction typically showing stronger associations than parental rejection (McLeod et al., 2007; Pinquart, 2017). A meta-analytic review focusing on parental control further reported a medium association between observed parental control and child anxiety (Van Der Bruggen et al., 2008). These patterns align with developmental models in which autonomy restriction reduces perceived control and mastery, while rejection may sensitize adolescents to social threat, both of which are implicated in anxiety maintenance (Nanda et al., 2012; Niditch and Varela, 2012; Fox et al., 2022). Parental psychological control has likewise been associated with maladaptive outcomes in Chinese students, and physical activity has moderated some of these associations (Wang et al., 2025).

### 1.3 Resilience as a mediating mechanism

Resilience offers a plausible pathway linking PA and parenting to anxiety (Liu et al., 2024; Cui et al., 2025). Resilience is commonly defined as positive adaptation despite adversity and is increasingly treated as a dynamic process supported by both individual assets and ecological resources, including supportive caregiving and opportunities for autonomy and mastery (Tsai et al., 2017; Luo D. et al., 2025). It has often been measured with the Connor–Davidson Resilience Scale (CD-RISC) (Connor and Davidson, 2003). In Chinese research, a three-component structure—tenacity, strength, and optimism—has been widely reported and applied (Yu and Zhang, 2007; Shi et al., 2016). In a recent systematic review and meta-analysis, higher resilience was associated with better mental health in adolescents and young adults, including lower anxiety (Wu et al., 2025b).

Resilience has also been implicated as a mediator. Adolescent path models suggested that resilience partially mediates associations between PA and mental health, and meta-analytic structural equation modelling similarly indicated indirect effects of PA on negative mental health indicators through resilience (Cui et al., 2025; Shi et al., 2025). Among college students, psychological resilience has been found to mediate and moderate the association between physical activity and negative emotion (Zuo et al., 2025). Mediation of family-environment effects has also been observed, with resilience bridging family satisfaction and lower anxiety in Chinese adolescents (Zhang et al., 2023).

### 1.4 Theoretical framework

The associations examined here can be organized within a developmental–ecological account of adaptation. Bronfenbrenner’s ecological systems perspective situates adolescent development within nested contexts, so that proximal settings such as the family and daily health behaviours shape psychological functioning (Bronfenbrenner and Morris, 2006). Within this perspective, the resilience framework in developmental psychopathology treats positive adaptation as the product of promotive and protective resources that operate across contexts rather than as a fixed trait (Masten, 2001; Masten and Barnes, 2018). Physical activity and supportive parenting can be read as two such resources. The stress-buffering and cross-stressor adaptation accounts hold that regular physical activity improves the capacity to regulate physiological and emotional responses to stressors, which lowers vulnerability to anxiety (Silverman and Deuster, 2014). Self-determination theory offers a complementary reading of parenting: warmth supports the needs for relatedness and competence, whereas rejection and intrusive overprotection frustrate relatedness and autonomy, with consequences for self-regulation and distress (Ryan and Deci, 2000). These accounts converge on resilience as a mediating capacity through which physical activity and parenting are linked to social anxiety, and they motivate the integrated model tested below.

### 1.5 The present study

**Figure 1:**
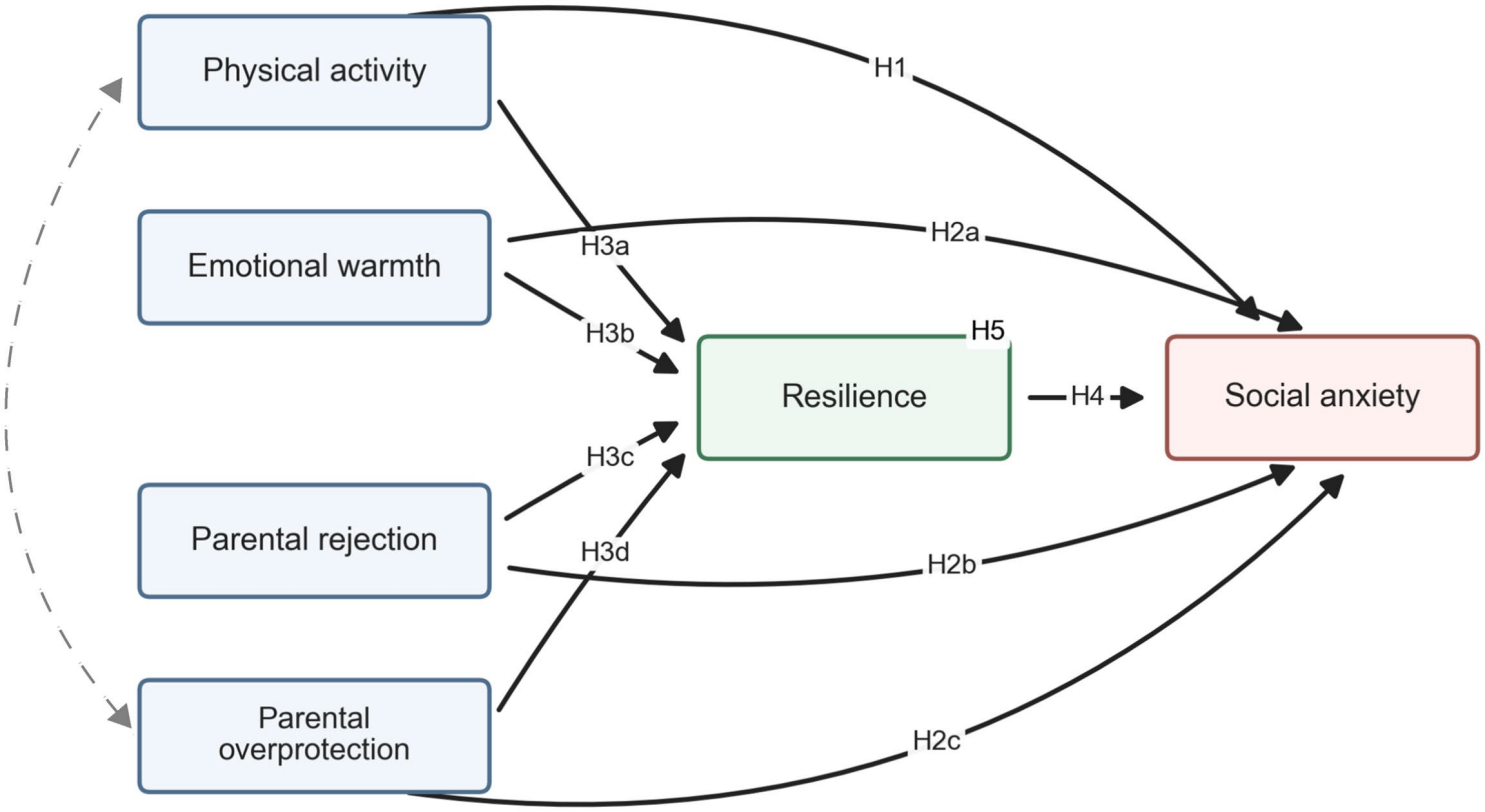
Hypothesized model. Single-headed arrows represent the hypothesized direct and mediated associations (H1–H5); dashed double-headed arrows represent estimated covariances among the exogenous predictors. PA, physical activity.

Despite these advances, integrated tests that model PA and specific parenting dimensions simultaneously, while estimating resilience as a multidimensional mediator, remain uncommon. The present study therefore tested a unified model in which PA and parenting style (rejection, emotional warmth, overprotection) predict adolescent social anxiety both directly and indirectly through resilience (tenacity, strength, optimism). Because parenting practices can shape adolescents’ opportunities to be active, covariances among the exogenous predictors were modelled. On the basis of the evidence reviewed above and the developmental–ecological framework, the following hypotheses were tested (Figure 1):

*H1*. Physical activity is negatively associated with social anxiety.

*H2*. Parenting style is associated with social anxiety, such that emotional warmth is negatively associated (H2a), whereas rejection (H2b) and overprotection (H2c) are positively associated with social anxiety.

*H3*. Physical activity (H3a) and emotional warmth (H3b) are positively, and rejection (H3c) and overprotection (H3d) are negatively, associated with resilience.

*H4*. Resilience is negatively associated with social anxiety.

*H5*. Resilience mediates the associations of physical activity and parenting style with social anxiety.

This study contributes by integrating modifiable behavioural and family determinants within a single mediation framework and by modelling resilience with interpretable components. The resulting evidence may help align movement-based prevention with parenting-focused strategies to strengthen adolescents’ adaptive capacities during a sensitive developmental window.

## 2 Materials and methods

### 2.1 Participants and procedure

Participants were 1,094 adolescents recruited from primary, junior high, and high schools in Zhuhai, Guangdong Province, China. Cluster sampling was applied at the level of intact classes within the participating schools, and the questionnaires were administered online in 2023, after ethics approval. Students in the selected classes who were present on the survey day and provided complete responses were included; no participants were excluded. The mean age was 15.38 years (SD = 1.41), and 536 participants (49.0%) were boys. By school stage, 470 (43.0%) attended primary school, 468 (42.8%) junior high school, and 156 (14.2%) high school. The study was conducted in accordance with the Declaration of Helsinki and was reviewed and approved by the Academic Ethics Committee (Institutional Review Board) of the School of Teacher Education, Northwest Minzu University (approval number NWNU-EDU-IRB-2023-0316; 16 March 2023). Before participation, informed consent was obtained electronically from each participant and from a parent or legal guardian; participation was voluntary, respondents could withdraw at any stage without penalty, and no identifying personal data were collected. The participant flow is summarized in Supplementary Figure S1.

### 2.2 Physical activity

The Chinese version of the Physical Activity Rating Scale, third revision (PARS-3), was used to assess physical activity. The scale includes three components: exercise intensity, duration of each session, and weekly exercise frequency. Each component is rated on a three-point scale, with 1 indicating light intensity or short duration, 2 indicating a moderate level, and 3 indicating high intensity or long duration. Total scores range from 0 to 100, with higher scores reflecting greater physical activity. The Chinese PARS-3 has shown good reliability and validity for assessing exercise load (Fang et al., 2025). In the present study, the scale demonstrated acceptable internal consistency (Cronbach’s *α* = 0.774).

### 2.3 Social anxiety

The Chinese version of the Social Anxiety Scale for Children (SASC) was used to assess social anxiety. The scale contains 10 items and covers two domains: fear of negative evaluation and social avoidance/distress. Each item is rated on a three-point scale, where 0 indicates “never,” 1 indicates “sometimes,” and 2 indicates “always.” Total scores range from 0 to 20, with higher scores indicating greater social anxiety. The Chinese SASC has shown good reliability and validity in child populations (La Greca et al., 1988). In the present study, the scale demonstrated good internal consistency (Cronbach’s *α* = 0.806).

### 2.4 Resilience

The Chinese version of the Connor–Davidson Resilience Scale (CD-RISC) was used to assess resilience. The scale includes 25 items and measures three dimensions: tenacity, strength, and optimism. Responses are rated on a five-point Likert scale ranging from 0 to 4, where 0 indicates “completely incorrect,” 1 indicates “rarely correct,” 2 indicates “sometimes correct,” 3 indicates “often correct,” and 4 indicates “almost always correct” (Yu and Zhang, 2007). Total scores range from 0 to 100, with higher scores indicating greater resilience. The Chinese CD-RISC has been widely validated in adolescent samples and has shown strong psychometric properties. In the present study, internal consistency was excellent (Cronbach’s *α* = 0.946). Confirmatory factor analysis supported the measurement model (*χ*^2^/df = 1.144, RMSEA = 0.011, CFI = 0.998, TLI = 0.997, IFI = 0.998).

### 2.5 Parenting style

Parenting style was assessed using the short form of the Egna Minnen Barndoms Uppfostran (S-EMBU) scale (Arrindell et al., 1999), which includes 23 items measuring three dimensions: rejection, overprotection, and emotional warmth. Participants rated their responses on a four-point scale: “1 = never,” “2 = occasionally,” “3 = often,” and “4 = very often.” Higher scores in each dimension reflect more frequent use of the respective parenting style. The Chinese S-EMBU has been validated for adolescent populations in China, with satisfactory reliability and validity. In this study, the Cronbach’s *α* for the scale was 0.771. Confirmatory factor analysis supported the three-factor model (*χ*^2^/df = 1.152, RMSEA = 0.012, CFI = 0.998, TLI = 0.997, IFI = 0.998).

### 2.6 Statistical analysis

Descriptive statistics were computed for all study variables. Group differences across the low-, middle-, and high-anxiety groups were examined using chi-square tests for categorical variables and one-way analysis of variance for continuous variables, followed by pairwise comparisons where appropriate. Scale reliability was assessed using Cronbach’s *α*. Confirmatory factor analysis was conducted to evaluate the measurement structure of the latent constructs. Composite reliability (CR) and average variance extracted (AVE) were calculated to assess construct reliability and convergent validity, with CR > 0.70 and AVE > 0.50 considered acceptable.

Structural equation modelling (SEM) was used to examine the relationships among physical activity, parenting style, resilience, and social anxiety (Figure 1). Resilience was specified as a latent construct measured by its three dimensions (tenacity, strength, and optimism), and social anxiety was specified as a latent construct indicated by its 10 items. In the structural model, physical activity, entered as its observed PARS-3 total score, and the three parenting dimensions (rejection, emotional warmth, and overprotection), each modelled as a latent factor from its S-EMBU items, were specified as exogenous predictors, and resilience and social anxiety were treated as endogenous variables. Direct paths were estimated from each predictor to resilience and to social anxiety, and a path from resilience to social anxiety was included to test the hypothesized mediation. Covariances among the exogenous predictors were estimated, and measurement errors and residual terms were specified for the indicators and endogenous constructs. Standardized coefficients are reported to aid interpretation. Indirect effects were tested with bias-corrected bootstrapping based on 2,000 resamples, with a 95% confidence interval excluding zero taken to indicate a significant effect.

The adequacy of the sample size was considered in relation to model complexity. With 1,094 cases, the ratio of observations to freely estimated parameters exceeded the commonly recommended minimum of 10:1, and a sample of this size is well above the range at which maximum-likelihood estimates and their standard errors are stable for models of this complexity (Kline, 2016; Wolf et al., 2013). For a model of these degrees of freedom, a sample of this magnitude also provides high power to detect misspecification using the RMSEA-based test of close fit (MacCallum et al., 1996). The sample therefore exceeded the minimum requirements for stable estimation and adequate statistical power in models of this size.

## 3 Results

### 3.1 Descriptive statistics and group differences

In the full sample, 536 participants (49.0%) were boys. The proportion of boys differed across social-anxiety groups (*χ*^2^ = 6.84, *p* = 0.033), being lower in the high-anxiety group than in the low-anxiety group (38.2% vs. 52.2%, *p* = 0.021). Age differed across groups (*F* = 14.62, *p* < 0.001); adolescents in the high-anxiety group were older than those in the low-anxiety group (15.82 ± 1.47 vs. 15.12 ± 1.35 years, *p* < 0.001). Group composition by school stage also differed, with a significant difference between the primary-school and junior-high groups (*p* = 0.008) but not between the junior-high and high-school groups (*p* = 0.112). Descriptive statistics and group comparisons are summarized in Table 1.

**Table 1:** Demographic characteristics of the sample across social-anxiety groups.

Variable	Whole sample	Low anxiety (*n* = 536)	Middle anxiety (*n* = 372)	High anxiety (*n* = 186)	Test	Key comparison
Boys, *n* (%)	536 (49.0)	280 (52.2)	185 (49.7)	71 (38.2)	*χ^2^* = 6.84, *p* = 0.033	Low vs. high: *p* = 0.021
Age (years), *M* ± SD	15.38 ± 1.41	15.12 ± 1.35	15.44 ± 1.40	15.82 ± 1.47	*F* = 14.62, *p* < 0.001	Low vs. high: *p* < 0.001
Primary school, *n* (%)	470 (43.0)	276 (51.5)	112 (30.1)	82 (44.1)	–	Primary vs. junior: *p* = 0.008
Junior high, *n* (%)	468 (42.8)	205 (38.2)	192 (51.6)	71 (38.2)	–	Junior vs. high: *p* = 0.112
High school, *n* (%)	156 (14.2)	55 (10.3)	68 (18.3)	33 (17.7)	–	–

Percentages are column-wise (computed within each anxiety group); for sex, the value is the percentage of boys. *χ*^2^, chi-square test; *F*, one-way ANOVA. Cell counts sum to the group sizes and to the school-stage totals.

### 3.2 Reliability and convergent validity of the measurement model

Table 2 presents the composite reliability (CR) and average variance extracted (AVE) for the latent constructs. Rejection, emotional warmth, and overprotection showed satisfactory reliability and convergent validity, with CR values of 0.878, 0.909, and 0.910 and AVE values of 0.546, 0.588, and 0.559, respectively. The three resilience dimensions also performed well: tenacity (CR = 0.943, AVE = 0.559), strength (CR = 0.924, AVE = 0.602), and optimism (CR = 0.850, AVE = 0.586). Social anxiety reached acceptable composite reliability (CR = 0.807), but its AVE (0.296) fell below the conventional 0.50 threshold for convergent validity. Most constructs therefore met the commonly accepted criteria of CR > 0.70 and AVE > 0.50, indicating generally adequate convergent validity across the measurement model. Because the SASC is a well-established scale and its composite reliability was acceptable (CR = 0.807), all 10 items were retained; the lower AVE for social anxiety is addressed in the Limitations.

**Table 2:** Composite reliability and average variance extracted of the latent constructs.

Construct	No. of items	CR	AVE
Rejection	6	0.878	0.546
Emotional warmth	7	0.909	0.588
Overprotection	8	0.910	0.559
Tenacity	13	0.943	0.559
Strength	8	0.924	0.602
Optimism	4	0.850	0.586
Social anxiety	10	0.807	0.296

CR, composite reliability; AVE, average variance extracted. CR > 0.70 and AVE > 0.50 indicate adequate convergent validity. The AVE for social anxiety is below 0.50 and is discussed in the Limitations.

### 3.3 Structural model and path coefficients

**Figure 2:**
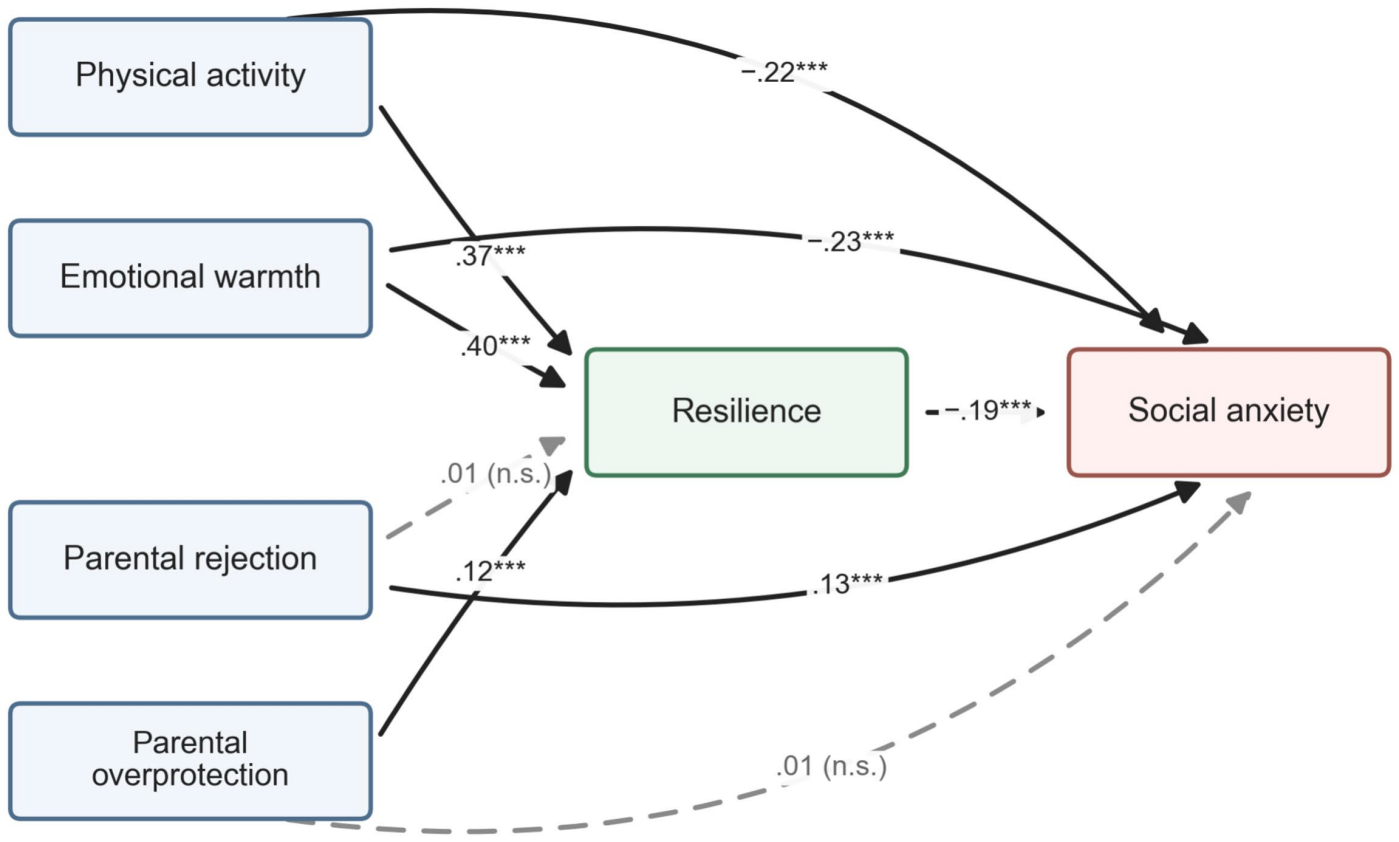
Structural model with standardized path coefficients. Solid lines indicate significant paths and dashed lines nonsignificant paths. n.s., nonsignificant; ^***^p < 0.001.

Table 3 and Figure 2 report the structural paths. Physical activity was positively associated with resilience (*B* = 0.2223, SE = 0.0163, *β* = 0.369, CR = 13.658, *p* < 0.001). Emotional warmth also showed a positive association with resilience (*B* = 1.3649, SE = 0.0932, *β* = 0.397, CR = 14.643, *p* < 0.001), as did overprotection (*B* = 0.3965, SE = 0.0902, *β* = 0.120, CR = 4.397, *p* < 0.001). The path from rejection to resilience was not significant (*B* = 0.0355, SE = 0.1148, *β* = 0.008, CR = 0.309, *p* = 0.757).

**Table 3:** Path coefficients of the structural model and significance of the estimated parameters.

Path	*B*	SE	*β*	CR (*t*)	*p*
Physical activity → Resilience	0.2223	0.0163	0.369	13.658	<0.001
Emotional warmth → Resilience	1.3649	0.0932	0.397	14.643	<0.001
Rejection → Resilience	0.0355	0.1148	0.008	0.309	0.757
Overprotection → Resilience	0.3965	0.0902	0.120	4.397	<0.001
Physical activity → Social anxiety	−0.0264	0.0037	−0.218	−7.186	<0.001
Emotional warmth → Social anxiety	−0.1612	0.0212	−0.234	−7.589	<0.001
Rejection → Social anxiety	0.1089	0.0239	0.125	4.552	<0.001
Overprotection → Social anxiety	0.0034	0.0190	0.005	0.178	0.859
Resilience → Social anxiety	−0.0379	0.0063	−0.189	−6.002	<0.001

*B*, unstandardized coefficient; SE, standard error; *β*, standardized coefficient; CR (*t*), critical ratio (estimate/SE). Non-significant paths are shown with their exact *p* values.

For social anxiety, physical activity showed a significant negative association (*B* = −0.0264, SE = 0.0037, *β* = −0.218, CR = −7.186, *p* < 0.001), as did emotional warmth (*B* = −0.1612, SE = 0.0212, *β* = −0.234, CR = −7.589, *p* < 0.001). Rejection was positively associated with social anxiety (*B* = 0.1089, SE = 0.0239, *β* = 0.125, CR = 4.552, *p* < 0.001), whereas the direct effect of overprotection was not significant (*B* = 0.0034, SE = 0.0190, *β* = 0.005, CR = 0.178, *p* = 0.859). Resilience was negatively associated with social anxiety (*B* = −0.0379, SE = 0.0063, *β* = −0.189, CR = −6.002, *p* < 0.001).

### 3.4 Mediation (indirect effects)

Bias-corrected bootstrapping (2,000 resamples) was used to test the indirect effects. Physical activity had a significant indirect effect on social anxiety through resilience (indirect effect = −0.0084, Boot SE = 0.0018, 95% CI [−0.0121, −0.0049]). Emotional warmth also showed a significant indirect effect (indirect effect = −0.0518, Boot SE = 0.0113, 95% CI [−0.0741, −0.0302]), as did overprotection (indirect effect = −0.0150, Boot SE = 0.0045, 95% CI [−0.0249, −0.0069]). The indirect effect of rejection through resilience was not significant, because its confidence interval included zero (indirect effect = −0.0013, Boot SE = 0.0043, 95% CI [−0.0101, 0.0069]). Results are reported in Table 4.

**Table 4:** Bootstrap results for the indirect (mediation) effects on social anxiety.

Indirect path	Indirect effect	Boot SE	95% LLCI	95% ULCI
Physical activity → Resilience → Social anxiety	−0.0084	0.0018	−0.0121	−0.0049
Emotional warmth → Resilience → Social anxiety	−0.0518	0.0113	−0.0741	−0.0302
Rejection → Resilience → Social anxiety	−0.0013	0.0043	−0.0101	0.0069
Overprotection → Resilience → Social anxiety	−0.0150	0.0045	−0.0249	−0.0069

Based on 2,000 bootstrap resamples. Boot SE, bootstrap standard error; LLCI/ULCI, lower/upper limit of the 95% confidence interval. An indirect effect is significant when the 95% CI excludes zero.

Table 5 summarizes the direct, indirect, and total effects. Physical activity and emotional warmth each combined a negative direct effect with a negative indirect effect through resilience, and emotional warmth showed the largest total protective effect (total effect = −0.2130). Rejection had a positive direct effect and a small, nonsignificant indirect effect, leaving a positive total effect (0.1075) consistent with a risk factor. Overprotection combined a small, nonsignificant direct effect with a negative indirect effect through resilience, producing a slightly negative total effect (−0.0117). The indirect effects operated primarily through resilience.

**Table 5:** Direct, indirect, and total effects of the predictors on social anxiety.

Predictor	Direct effect	Indirect effect	Total effect
Physical activity	−0.0264	−0.0084	−0.0348
Emotional warmth	−0.1612	−0.0518	−0.2130
Rejection	0.1089	−0.0013	0.1075
Overprotection	0.0034	−0.0150	−0.0117

The dependent variable is social anxiety. Indirect effects operate through resilience; the total effect is the sum of the direct and indirect effects.

## 4 Discussion

Physical activity positively predicted resilience (*β* = 0.369) and negatively predicted social anxiety (*β* = −0.218), and resilience was negatively associated with social anxiety (*β* = −0.189). Parental emotional warmth showed a positive association with resilience (*β* = 0.397) and a negative association with social anxiety (*β* = −0.234), yielding the largest overall protective effect. Parental rejection was positively associated with social anxiety (*β* = 0.125), whereas its path to resilience was not significant (*β* = 0.008). Parental overprotection was positively associated with resilience (*β* = 0.120), but its direct path to social anxiety was not significant (*β* = 0.005). Bootstrapping indicated that resilience significantly mediated the effects of physical activity, emotional warmth, and overprotection on social anxiety, with no significant mediation for rejection. The pattern suggests a structured profile of risk and protection in which physical activity and family warmth operate as protective channels, rejection functions as a direct risk channel, and resilience serves as a shared mechanism.

### 4.1 Physical activity, resilience, and social anxiety

The negative association between physical activity and social anxiety is consistent with prior evidence. Systematic reviews and meta-analyses of prospective cohort studies have linked higher physical activity to a reduced risk of subsequent anxiety symptoms and disorders, and meta-analyses of randomized controlled trials in children and adolescents have shown that physical activity interventions can reduce anxiety, although study quality has been variable and evidence from clinical samples remains limited. Given the significant path from physical activity to resilience, it is plausible that physical activity strengthens resilience by improving stress-coping capacity, promoting behavioural activation, and increasing self-efficacy. This reading aligns with mediation frameworks in which physical activity improves mental health partly through psychological resources (Zuo et al., 2025).

### 4.2 Emotional warmth as a protective family factor

The links of parental emotional warmth with higher resilience and lower social anxiety are in line with the literature (Zhao et al., 2024; Howard et al., 2025). Developmental and clinical accounts treat warm, supportive parenting as a key environmental resource (Lin S. C. et al., 2024; Sun et al., 2025) that fosters security, supports emotion regulation, and strengthens adaptive systems and resilience (Zhao et al., 2024; Zeng et al., 2025). The present findings extend this work by showing that emotional warmth was related to lower social anxiety not only directly but also indirectly through increased resilience, consistent with reports in which resilience mediates associations between family functioning and anxiety or depression (Ao et al., 2025; Li et al., 2025).

### 4.3 Parental rejection as a direct risk factor

The direct effect of parental rejection on higher social anxiety supports a negative-parenting risk pathway and is consistent with meta-analytic findings on rejection or hostility and child anxiety. Neither the path from rejection to resilience nor the indirect effect through resilience was significant. This pattern suggests that rejection may operate through mechanisms closer to the anxiety phenotype, such as heightened threat sensitivity, negative-evaluation expectations, or social avoidance, which may not be captured by the resilience facets of tenacity, strength, and optimism. A statistical reading is also possible: when emotional warmth and overprotection are modelled together, the unique variance in rejection available to explain resilience is reduced, so rejection can remain salient for anxiety while showing a nonsignificant association with resilience. This is broadly consistent with evidence that parenting explains a modest share of variance in child anxiety.

### 4.4 Parental overprotection

The absence of a significant direct effect of overprotection on social anxiety, alongside a positive association with resilience and a small negative indirect effect, is informative (de Roo et al., 2022; Huang, 2025). Previous meta-analyses reported a medium association between overprotection and child anxiety (McLeod et al., 2007; de Roo et al., 2022); in the present model, the nonsignificant direct path may reflect the joint inclusion of warmth, rejection, and overprotection (Pinquart, 2017). After controlling for warmth and rejection, the residual component of overprotection may have reflected structured support or protective investment, which could relate to higher resilience (Lee et al., 2016; He et al., 2025), while its autonomy-limiting component may have been absorbed by shared variance with the other dimensions (Li et al., 2019; Chyung et al., 2022). This explanation is tentative and should be tested directly. Future work would benefit from separating behavioural from psychological control, modelling maternal and paternal dimensions separately, and using longitudinal designs (Gao et al., 2026; Scowen, 2025).

### 4.5 Resilience as a mediating mechanism

The negative association between resilience and social anxiety accords with resilience theory and empirical evidence (Yu and Zhang, 2007; Luo D. et al., 2025, Luo S. et al., 2025). The CD-RISC conceptualizes resilience as a measurable set of capacities linked to psychological adaptation (Connor and Davidson, 2003), and in Chinese samples the three-factor structure of tenacity, strength, and optimism has been widely applied (Yu and Zhang, 2007; Wu et al., 2025b). Systematic reviews indicate that resilience is associated with better mental health and is moderately negatively correlated with anxiety (Luo D. et al., 2025, Luo S. et al., 2025). Building on this, the present results indicate partial mediation by resilience for the protective effects of physical activity and emotional warmth (Lin H. et al., 2024; Wu et al., 2025b), consistent with pathway models in which physical activity contributes to mental health partly through increased resilience.

## 5 Implications

These findings indicate that adolescent social anxiety is shaped by both behavioural and family pathways. Physical activity and parental emotional warmth were associated with lower social anxiety, and these links were partly explained by resilience. Parental rejection showed a direct positive association with social anxiety, whereas overprotection was related to anxiety mainly through an indirect pathway. This pattern highlights resilience as an important mechanism linking external experiences to mental-health outcomes, and it points to integrated prevention that combines physical activity promotion, supportive parenting, and resilience training.

The study also adds nuance to the parenting literature. Emotional warmth emerged as the strongest protective factor, whereas rejection functioned mainly as a direct risk factor. Parenting dimensions may therefore influence anxiety through different routes and should be examined separately rather than as a single composite.

## 6 Limitations

Several limitations should be acknowledged. First, the cross-sectional design precludes causal inference and does not allow the temporal ordering of physical activity, parenting style, resilience, and social anxiety to be determined. Second, all variables were assessed by self-report, which may have introduced common-method bias, recall error, and subjective distortion. Third, although most constructs showed acceptable psychometric properties, the relatively low AVE for social anxiety suggests that this construct was measured with lower precision than the others. Fourth, the sample was drawn from a specific school-based population in China, which may limit generalizability to other age groups, clinical populations, or cultural contexts. Finally, other relevant influences, such as socioeconomic conditions, parental mental health, peer relationships, and academic stress, were not modelled. The present findings should therefore be read as evidence of theoretically meaningful associations within a defined context, and longitudinal, multi-informant, and cross-cultural studies are needed to strengthen causal interpretation.

## 7 Conclusion

This study provides an integrated account of how physical activity, parenting style, and resilience are associated with adolescent social anxiety. Physical activity and parental emotional warmth were both linked to lower social anxiety and higher resilience, whereas parental rejection was associated with higher social anxiety. Parental overprotection showed no direct association with social anxiety but was indirectly related to lower anxiety through resilience. Resilience emerged as a key mediator, carrying the effects of physical activity, emotional warmth, and overprotection on social anxiety. These findings suggest that adolescent social anxiety is shaped by both behavioural and family pathways, with resilience serving as an important psychological mechanism, and they support intervention strategies that combine physical activity promotion, supportive parenting, and resilience enhancement (Wang et al., 2024a; Howard et al., 2025; Wu et al., 2025a).

## Data Availability

The original contributions presented in the study are included in the article/supplementary material, further inquiries can be directed to the corresponding author.
